# An esculentin-1 homolog from a dark-spotted frog (*Pelophylax nigromaculatus*) possesses antibacterial and immunoregulatory properties

**DOI:** 10.1186/s12917-024-04013-y

**Published:** 2024-04-27

**Authors:** Jie Chen, Ci-Gang Yu, Min-Min Zhou, Gao-Jian Zhang, Hai-Long Su, Guo-Hua Ding, Li Wei, Zhi-Hua Lin, Li Ma

**Affiliations:** 1 College of Ecology, Lishui University, Lishui, 323000 China; 2 Nanjing Institute of Environmental Sciences, Ministry of Ecology and Environment, Nanjing, 210042 China

**Keywords:** Antimicrobial peptides, Esculentin-1, Antibacterial activity, Immunoregulatory activity, Dark-spotted frog

## Abstract

**Background:**

Esculentin-1, initially discovered in the skin secretions of pool frogs (*Pelophylax lessonae*), has demonstrated broad-spectrum antimicrobial activity; however, its immunomodulatory properties have received little attention.

**Results:**

In the present study, esculentin-1 cDNA was identified by analysing the skin transcriptome of the dark-spotted frog (*Pelophylax nigromaculatus*). Esculentin-1 from this species (esculentin-1PN) encompasses a signal peptide, an acidic spacer peptide, and a mature peptide. Sequence alignments with other amphibian esculentins-1 demonstrated conservation of the peptide, and phylogenetic tree analysis revealed its closest genetic affinity to esculentin-1P, derived from the Fukien gold-striped pond frog (*Pelophylax fukienensis*). Esculentin-1PN transcripts were observed in various tissues, with the skin exhibiting the highest mRNA levels. Synthetic esculentin-1PN demonstrated antibacterial activity against various pathogens, and esculentin-1PN exhibited bactericidal activity by disrupting cell membrane integrity and hydrolyzing genomic DNA. Esculentin-1PN did not stimulate chemotaxis in RAW264.7, a murine leukemic monocyte/macrophage cell line. However, it amplified the respiratory burst and augmented the pro-inflammatory cytokine gene (TNF-α and IL-1β) expression in RAW264.7 cells.

**Conclusions:**

This novel finding highlights the immunomodulatory activity of esculentin-1PN on immune cells.

**Supplementary Information:**

The online version contains supplementary material available at 10.1186/s12917-024-04013-y.

## Background

Antimicrobial peptides (AMPs) are a ubiquitous group of peptides found in diverse plant and animal species. They serve as crucial components of the immune response against a spectrum of pathogenic microorganisms, including bacteria, parasites, fungi, and viruses [1]. These peptides, which are characterised by their short length, cationic charge, and amphiphilic nature, confer host protection through direct interactions with bacterial membrane-associated proteins, resulting in membrane disruption and subsequent bacterial death [2, 3]. Besides their antimicrobial activity, recent studies have elucidated the immunomodulatory properties of AMPs [4].

The dermal glands of amphibians belong to the genus *Rana*, a widely distributed group in the Ranidae family. They contain an abundance of AMPs [5], which are synthesised by the serous dermal glands and stored within granules alongside various other pharmacologically active peptides. Upon experiencing stress or physical injury, these granules are released onto the skin surface via holocrine secretion [6–10]. Notably, each frog species produces its own distinct repertoire of AMPs [11]. Based on their structural similarities, amphibian AMPs are categorised into various families, such as magainins, brevinins, ranacyclins, and esculentins [12].

Esculentin-1 was initially discovered in the skin secretion of pool frogs (*Pelophylax lessonae*) [13]. It was subsequently isolated from closely related species, such as Yunnanfu frog (*Odorrana grahami*) [14], Ishikawa’s frog (*Odorrana ishikawae*) [15], Sahara frog (*Pelophylax saharicus*) [16], crawfish frog (*Rana areolata*) [17], Florida gopher frog (*Lithobates capito*) [18], and Warszewitsch’s Frog (*Lithobates warszewitschii*) [18]. Other esculentin-1 peptides were identified using cDNA libraries derived from frog skin [19, 20]. There are 46 amino acids in esculetin-1, which belongs to a peptide class with a highly conserved amino acid sequence. In these peptides, the C-terminal loop is stabilized by a disulfide bridge, forming a heptapeptide ring [21, 22].

Esculentin-1 has two homologous counterparts, esculentin-1a and esculentin-1b, which demonstrate a sole discrepancy in amino acid composition at position 11 within their respective sequences [21]. In recent years, Esc(1–21) and Esc(1–18), which correspond to the initial 20 and 18 amino acids of native esculentin-1a and -1b, respectively, have been synthesised and demonstrated to possess a broad range of bactericidal activities [23, 24] against various bacterial strains, including *Staphylococcus aureus*, *Enterococcus faecalis*, *Escherichia coli*, and *Enterobacter cloacae* [20, 25–32]. Despite the antimicrobial activity of esculentin-1, it also exhibits insulinotropic and β-cell protective activities [33]. However, the immunomodulatory activity of esculentin-1 remains under-studied and requires further research.

The dark-spotted frog (*Pelophylax nigromaculatus*), a semi-aquatic pond species indigenous to East Asia, was effectively propagated using an artificial diet. Consequently, dark-spotted frogs have been extensively cultivated for human consumption in China, with official government authorization. Regrettably, substantial economic losses were incurred owing to bacterial infections [34]. Acknowledging the significance of AMPs in the innate immune response, this study aimed to identify a counterpart of the dark-spotted frog esculentin-1 (esculentin-1PN) and explore the antibacterial and immunoregulatory activities of its mature peptide.

## Results

### Identification and characterisation of esculentin-1PN

**Fig. 1:**
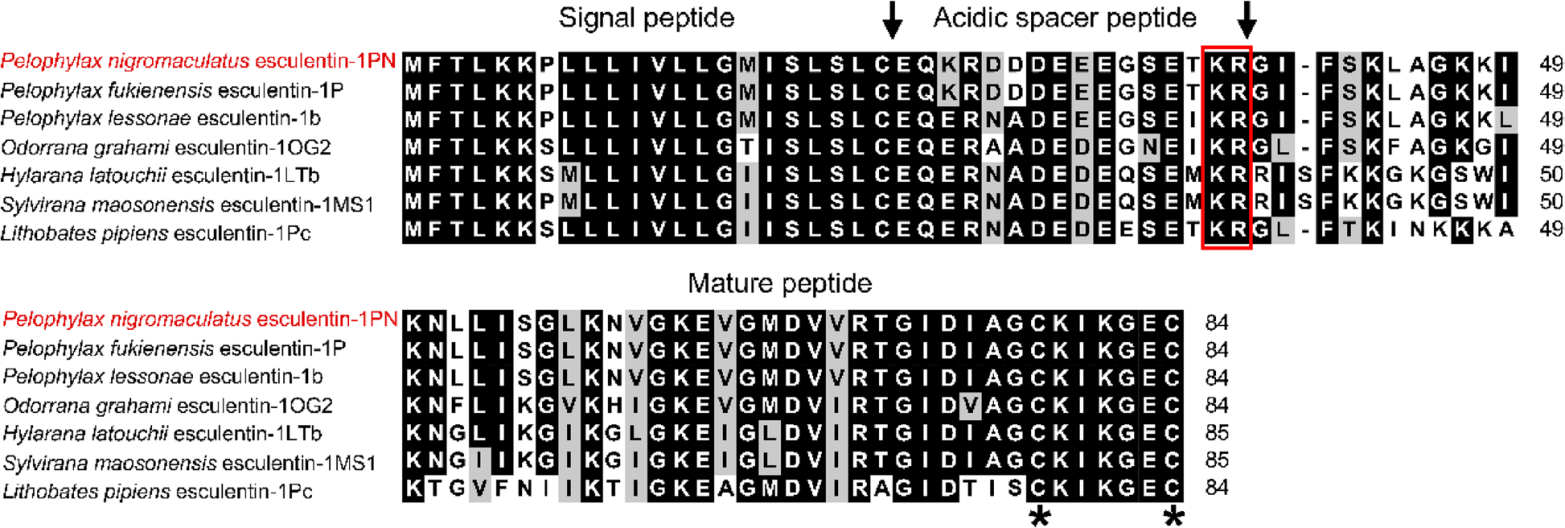
Multiple alignment of esculentin-1PN’s amino acid sequences and homologs. The threshold for shading was 70%; similar residues are marked with a grey shadow, identical residues with a black shadow, and alignment gaps with “-.” The alignment was accompanied by labels indicating the signal peptide, acidic spacer peptide, and mature peptide. An arrow (↓) marks the predicted cleavage site for either the signal peptide or the mature peptide. The KR-propeptide convertase processing site is enclosed within a red box. The presence of two conserved cysteine residues in the mature peptide is indicated by an asterisk (“*”)

**Fig. 2:**
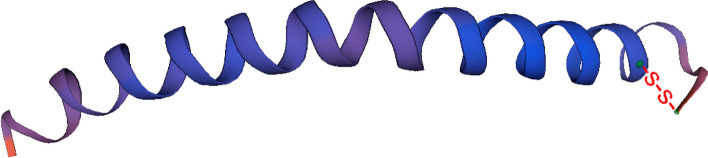
The structural model of esculentin-1PN. Molecular modelling was performed using SWISS-MODEL with the structure of esculentin-1b (PDB accession No. P84841.1.A) as the model. The disulfide bonds are denoted by the symbol -s–s-

The gene encoding the esculentin-1PN protein in dark-spotted frogs was successfully cloned, and its sequence was deposited in the GenBank Database (OR238914). The identified open reading frame within the esculentin-1PN gene spans 255 nucleotides and encodes 84 amino acids. The predicted molecular weight of this polypeptide is 9.16 kDa, with a theoretical isoelectric point (*p*I) of 8.84. The protein exhibits several characteristic structural features, including a putative signal peptide, an acidic spacer peptide, and a mature peptide. The signal peptide serves to guide the mature peptide to the extracellular compartment, while the acidic spacer peptide lacks a defined function. The mature peptide is predicted to have a molecular weight of 4.80 kDa, a *p*I of 9.63, and two conserved cysteine residues capable of forming a single pair of disulfide bonds (Fig. 1). The structure of the mature peptide is dominated by the alpha helices (Fig. 2).

**Fig. 3:**
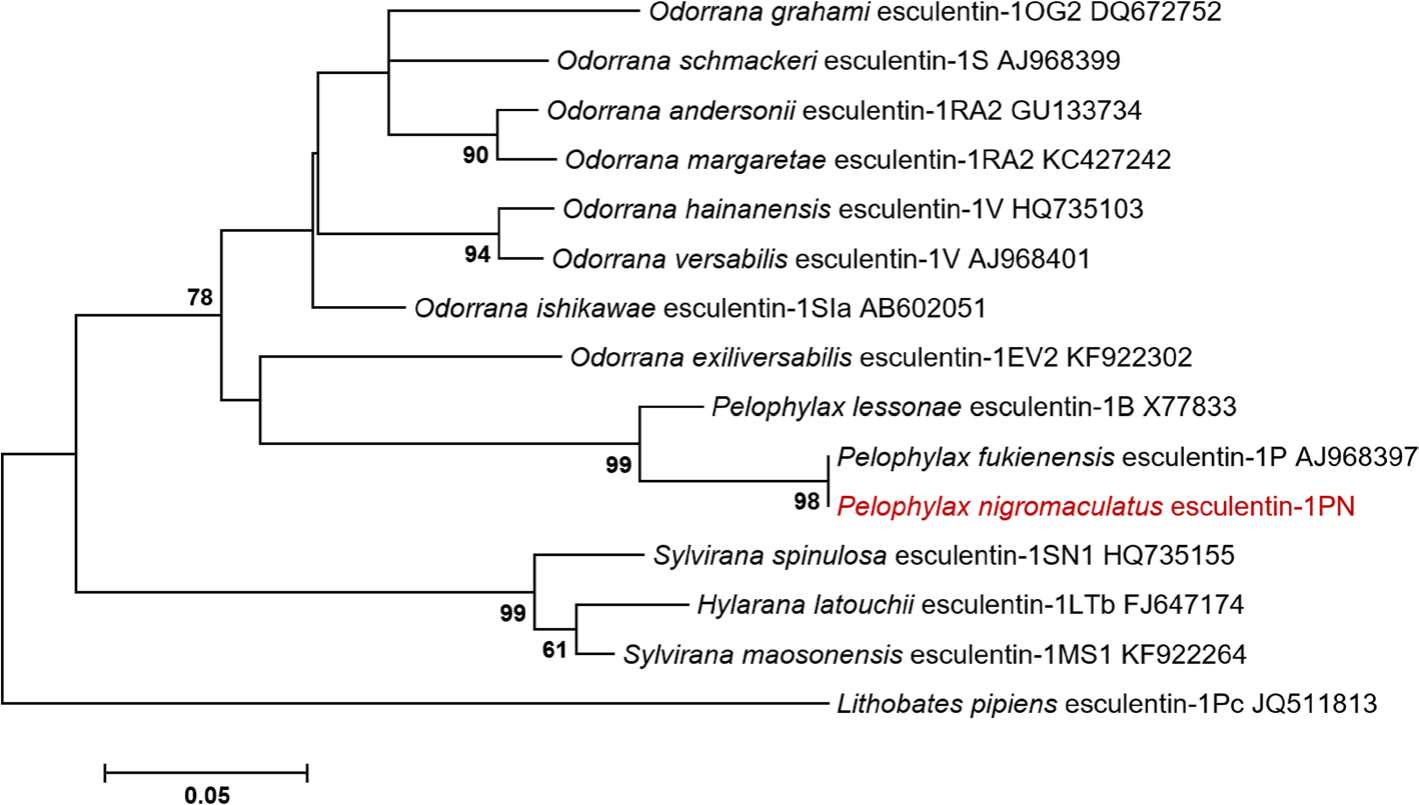
Phylogenetic reconstruction of esculentin-1’s amino acid sequences based on the neighbour-joining method. The values displayed at the forks represent the percentage of trees in which this particular grouping was observed after bootstrapping (1000 replicates), with values shown only when they exceed 60%

Phylogenetic tree analysis revealed that esculentin-1PN belonged to the Pelophylax esculentin-1 cluster and was most closely related to esculentin-1P derived from the Fukien gold-striped pond frog (*Pelophylax fukienensis*) (Fig. 3).

### Constitutive expression of the esculentin-1PN gene

**Fig. 4:**
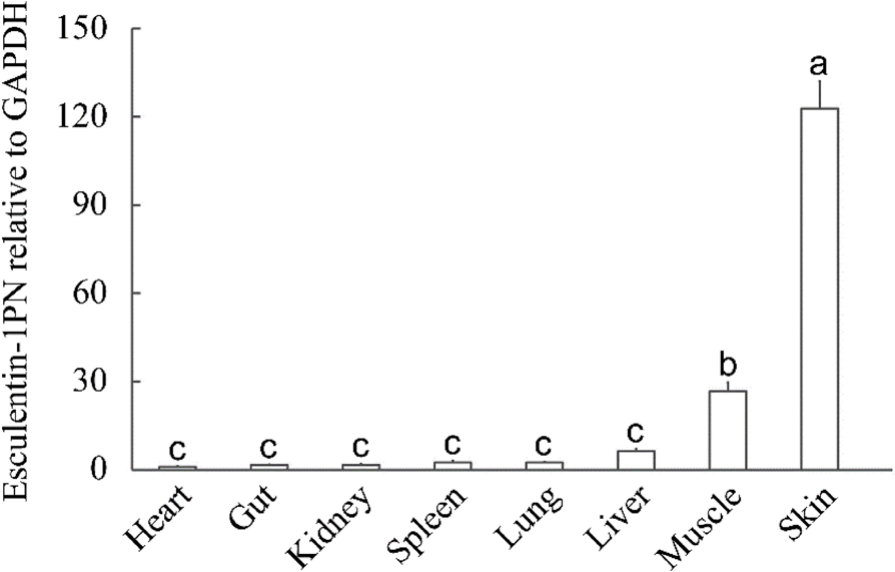
Constitutive expression of esculentin-1PN in various tissues of a dark-spotted frog, evaluated using qPCR. Expression levels of esculentin-1PN transcripts were standardised against the PnGAPDH gene. Data are presented as mean ± SEM (*n* = 4). Letters indicate significant differences as determined by one-way ANOVA (*P* < 0.05)

Constitutive expression of the esculentin-1PN gene was identified in all the dark-spotted frog tissues examined. The skin exhibited the highest expression level, followed by muscle, while the remaining tissues displayed comparatively lower expression levels (Fig. 4).

### Esculentin-1PN’s antibacterial activity

Esculentin-1PN exhibited varying levels of antibacterial activity, with the strongest antibacterial activity (minimal inhibitory concentration (MIC) = 6.25 μg/mL) observed against *V. anguillarum*, *E. coli*, and *S. saprophyticus*. The MIC of esculentin-1PN was 12.5 μg/mL against *C. freundii*, 25 μg/mL against *V. harveyi*, *V. alginolyticus*, and *P. aeruginosa*, and 50 μg/mL against *S. flexneri* and *L. monocytogenes*. The peptide did not exhibit any significant bactericidal activity against *A. hydrophila*, *P. mirabilis*, or *S. warneri*, as indicated in Table 1.

**Table 1:** Minimal inhibitory concentration (MIC) and minimum bactericidal concentration (MBC) values for esculentin-1PN against bacteria

Bacteria	Isolate/strain	MIC (μg/mL)	MBC (μg/mL)
gram-negative			
* Vibrio harveyi*	ATCC33866	25	200
* Vibrio alginolyticus*	ATCC17749	25	50
* Vibrio anguillarum*	ATCC19264	6.25	200
* Escherichia coli*	K12	6.25	50
* Citrobacter freundii*	ATCC43864	12.5	25
* Shigella flexneri*	ATCC12022	50	100
* Pseudomonas aeruginosa*	ATCC27853	25	100
* Aeromonas hydrophila*	ATCC7966	NT	NT
* Proteus mirabilis*	ATCC25933	NT	NT
gram-positive			
* Staphylococcus saprophyticus*	ATCC49907	6.25	25
* Staphylococcus warneri*	ATCC49454	NT	NT
* Listeria monocytogenes*	ATCC19115	50	100

*NT* no inhibition was detected at 100 μg/mL

### Impact of esculentin-1PN on Escherichia coli cell membrane integrity and gDNA

**Fig. 5:**
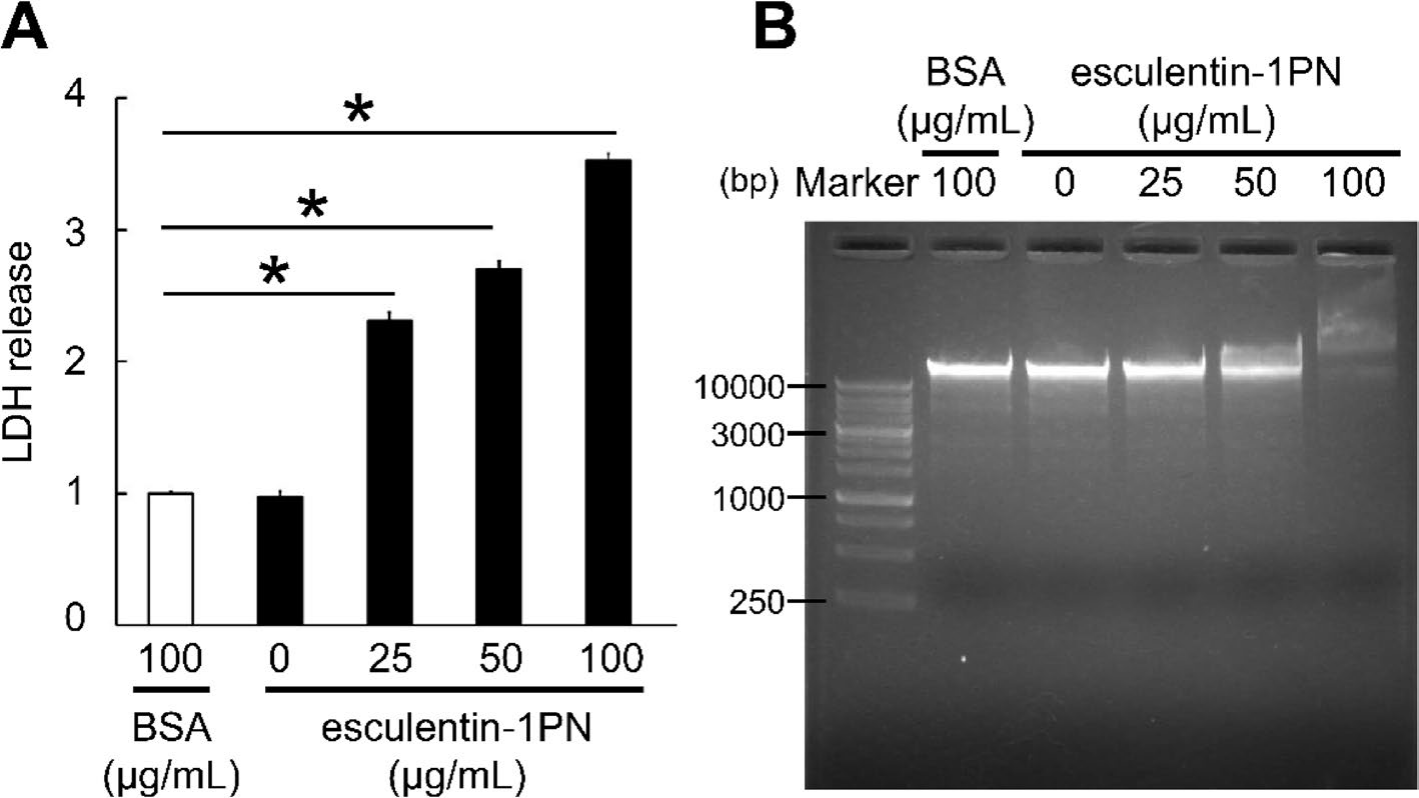
Impact of esculentin-1PN on the integrity of *Escherichia coli*’s cell membrane and genomic DNA. **A** Impact of esculentin-1PN on the integrity of the *E. coli* cell membrane. BSA was employed as a negative control. The release of lactate dehydrogenase (LDH) was measured and expressed as the fold change compared with the negative control, which was assigned a value of 1. **B** The hydrolytic activity of esculentin-1PN on the bacterial genomic DNA was assessed through electrophoresis. BSA was employed as a negative control. The data presented here represent the mean ± SEM (*n* = 4). Statistical analysis was performed using a one-way ANOVA. *P value < 0.05

The LDH assay demonstrated that esculentin-1PN had a deleterious effect on the integrity of the *E. coli* cell membrane. The most significant effect was observed at a concentration of 100 μg/mL, leading to a 3.53-fold increase in LDH release when compared to the control group (Fig. 5A). In Fig. 5B, it was observed through electrophoresis that there was a progressive decline in the intensity of gDNA bands, which was dependent on the concentration of esculentin-1PN.

### Effect of esculentin-1PN on RAW264.7 cell chemotaxis and respiratory burst

**Fig. 6:**
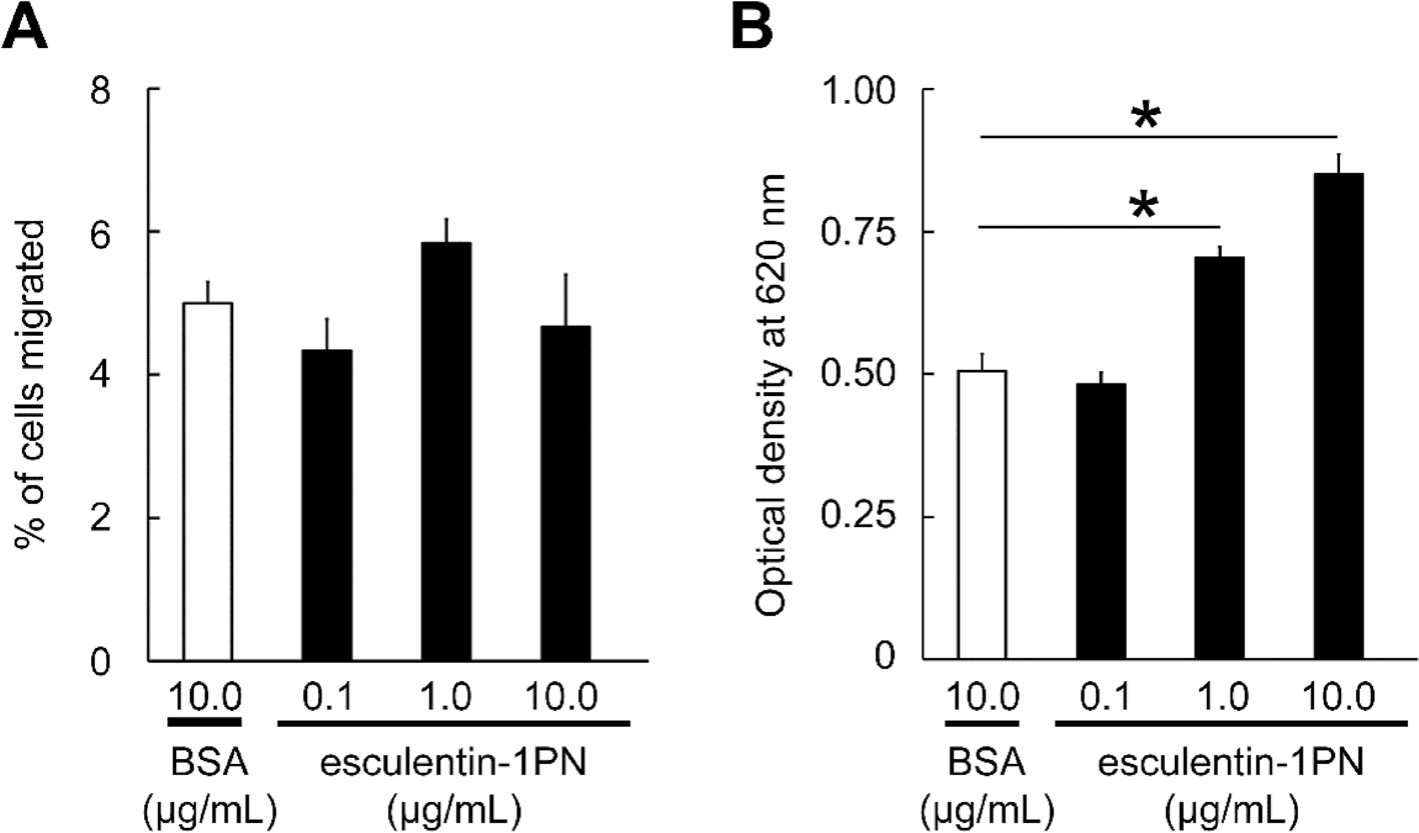
Impact of esculentin-1PN on the chemotaxis and respiratory burst of RAW264.7 cells. **A** Effect of esculentin-1PN on the migration of RAW264.7 cells. **B** Effect of esculentin-1PN on RAW264.7 cell respiratory burst. The data is presented as the mean ± SEM (*n* = 4). *P value < 0.05

The chemotaxis assay revealed that the percentage of RAW264.7 cell migration in the esculentin-1PN treatment group did not differ significantly from that in the BSA treatment group. This suggests that esculentin-1PN failed to induce chemotaxis in RAW264.7 cells (Fig. 6A). The effect of esculentin-1PN on respiratory burst in RAW264.7 cells was also investigated. The OD_620_ values recorded in the 1.0 and 10.0 μg/mL esculentin-1PN groups were 0.70 ± 0.02 and 0.85 ± 0.03, respectively, while the OD_620_ value observed in the BSA group was 0.51 ± 0.03. These findings provide evidence that esculentin-1PN significantly enhanced respiratory burst in RAW264.7 cells (Fig. 6B).

### Effect of esculentin-1PN on inflammatory cytokine expression in RAW264.7 cells

**Fig. 7:**
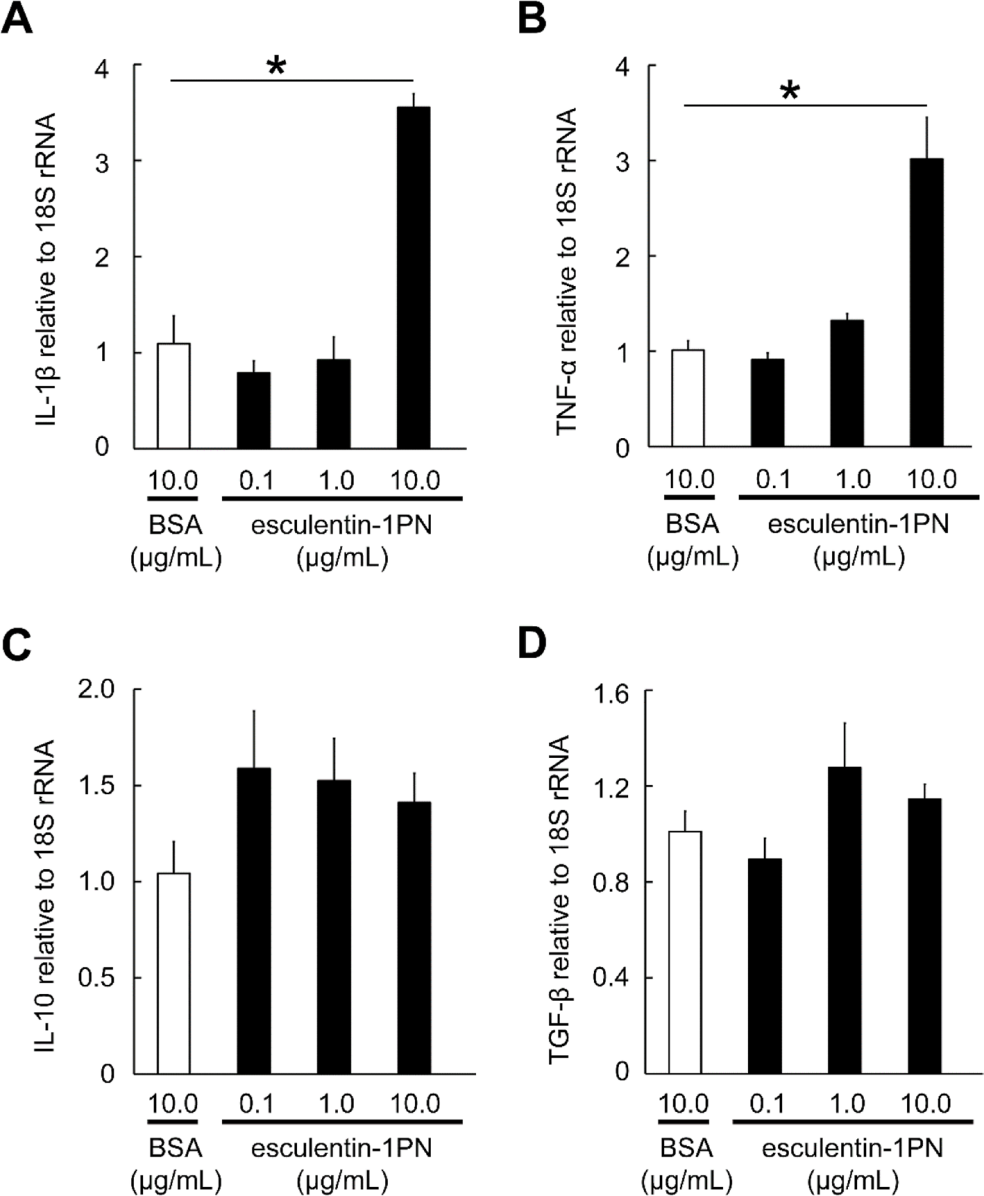
Effect of esculentin-1PN on inflammatory cytokine expression in RAW264.7 cells. Expression levels of IL-1β (**A**), TNF-α (B), IL-10 (**C**), and TGF-β (**D**) transcripts were normalised to those of the 18S rRNA gene. BSA was used as a control. Data are expressed as mean ± SEM (*n* = 4). *P value < 0.05

To evaluate the regulatory impact of esculentin-1PN on the expression of inflammatory cytokines, RAW264.7 cells were stimulated with varying concentrations of esculentin-1PN. Subsequently, a notable increase in the expression levels of IL-1β and TNF-α was observed in RAW264.7 cells, particularly following treatment with 10.0 μg/mL of esculentin-1PN (Fig. 7A,B). Conversely, the expression of IL-10 and TGF-β remained unaltered after esculentin-1PN treatment (Fig. 7C,D).

## Discussion

The conserved polypeptide esculentin-1, an amphibian AMP, was initially discovered in the skin secretions of pool frogs [13]. Extensive studies have been conducted on the antimicrobial properties of esculentin-1 in various amphibians [25–32]. However, knowledge of its immunoregulatory activity is limited. In this study, a cDNA encoding esculentin-1 was identified in the dark-spotted frogs, which comprises a signal peptide, an acidic spacer peptide, and a mature peptide. The C-terminal loop of the mature peptide is reinforced by a disulfide bridge, resulting in a heptapeptide ring. This structural characteristic is similar to that observed in other mature peptides of amphibian esculentin-1 [21, 22]. Both the esculentin-1PN precursor and the Fukien gold-striped pond frog esculentin-1P precursor were classified within the *Pelophylax* cluster. This identification offers crucial insights into the evolutionary connections between amphibian esculentin-1.

Esculentin-1 is constitutively expressed in the skin and other tissues of dark-spotted frogs. Amphibians have evolved various mechanisms to combat bacterial infections. One such mechanism involves the production of small compounds and peptides, such as esculentin-1, by the skin, which serve as a defence against pathogenic microorganisms [6, 35]. Previous studies have demonstrated that the mature esculentin-1 peptide, along with its two derivatives, Esc(1–21) and Esc(1–18), possess wide-ranging antimicrobial activity [20, 23–32]. Additionally, the combination of Esc(1–21) and colistin has been found to effectively inhibit growth and kill multidrug-resistant *Acinetobacter baumannii* strains [36]. Esculentin-1PN showed broad-spectrum antibacterial activities; it is most active against *V. alginolyticus*, *V. anguillarum*, and *E. coli*, as well as against *V. harveyi*, *S. saprophyticus*, *P. aeruginosa*, *C. freundii*, *S. flexneri*, and *L. monocytogenes*. Previous research has demonstrated that Esc(1–21) and Esc(1–18) possess the ability to disrupt the cell membranes of pathogenic bacteria [26, 27]. The findings from the LDH assay provide further evidence that esculentin-1PN can potentially impair bacterial cell membranes. Moreover, gDNA hydrolysis may play a role in its bactericidal mechanism, similar to other amphibian AMPs such as dark-spotted frog brevinin-2 [37], tiger frog (*Hoplobatrachus rugulosus*) cathelicidin [38], and Chong’an mustache toad (*Leptobrachium liui*) LEAP2 [39]. It is conceivable that the positively charged residues within esculentin-1PN facilitate the initial establishment of electrostatic interactions with the negatively charged DNA. Subsequently, there is a likelihood that a portion of esculentin-1PN gets inserted into the groove of the DNA molecule, thereby disrupting the double helix structure of bacterial gDNA and ultimately resulting in DNA strand breaks and degradation [40]. Nevertheless, the precise mechanism underlying DNA degradation remains to be further elucidated.

The immunomodulatory activity of esculentin-1 in amphibians requires further investigation. This study provides empirical evidence that esculentin-1PN does not exert any discernible influence on the chemotaxis of RAW264.7 cells. Nevertheless, it exerted a substantial dose-dependent enhancement of the respiratory burst of these cells. Leukocytes have been observed to effectively eliminate bacteria through phagocytosis, a process that involves the production of reactive oxygen species (ROS) and reactive nitrogen species (RNS). A respiratory burst, which signifies the oxygen-dependent eradication of bacteria, is a direct consequence of this phenomenon [41]. Notably, cathelicidin isolated from the tiger frog (*Hoplobatrachus rugulosus*) has been documented to exhibit immunomodulatory properties, notably augmenting the respiratory burst [38]. Upon activation by IFN-γ, murine macrophages exhibit heightened production of ROS and RNS, leading to the direct eradication of intracellular bacteria [42]. Furthermore, the increased levels of ROS and RNS in neutrophils serve as regulators, influencing the expression of essential cytokines and chemokines that significantly impact immune cell functions such as phagocytosis, bacterial clearance, and apoptosis [43]. The cytokine TNF-α is widely acknowledged as a key regulator in the control of ROS and RNS signaling, influencing the behavior of innate immune cells in both normal and inflammatory states [44]. Esculentin-1PN, due to its ability to stimulate respiratory burst, is hypothesised to impact ROS/RNS levels and thereby modulate the activities of RAW264.7 cells. Pro-inflammatory cytokines like TNF-α, IFN-γ, and IL-1β are crucial in initiating the innate immune response, with changes in their expression patterns in RAW264.7 cells serving as markers of cellular activation [45]. In the present study, treatment with esculentin-1PN enhanced TNF-α and IL-1β gene expression in RAW264.7 cells, indicating its potential role in stimulating monocytes/macrophages. Our results are consistent with previous findings that Chinese spiny frog β-defensin acts on RAW264.7 cells to upregulate the expression of TNF-α and IL-1β genes [46].

## Conclusion

In this study, we characterised an amphibian esculentin-1 gene derived from a dark-spotted frog. The chemically synthesised mature peptide, esculentin-1PN, demonstrated antibacterial activity against multiple bacterial strains. Additionally, esculentin-1PN exhibited noteworthy immunomodulatory activities by augmenting the respiratory burst and inducing expression of TNF-α and IL-1β in RAW264.7 cells. Nevertheless, additional research is warranted to comprehensively elucidate the underlying mechanisms.

## Methods

### Animals

The collection of all samples was conducted with due permission in compliance with the local license. The methods employed were executed in adherence to the pertinent guidelines and regulations stipulated in the ethics approval and consent to participate section, as well as with the endorsement of the Ethics Committee of Lishui University (Permit No. AREC-LSU202302–008) and ARRIVE guidelines.

Dark-spotted frogs weighing 80–100 g were procured from a farm in Lishui, China. These frogs were housed in 100-L tanks maintained at a temperature range of 23–25 °C. Before being utilised in the study, they were subjected to a commercial diet twice daily and allowed to acclimatise to laboratory conditions for 2 weeks. Experimental animals were managed according to China’s Experimental Animal Management Law, and Lishui University’s Ethics Committee approved all experiments.

### Esculentin-1PN cDNA sequencing analyses

The cDNA sequence of esculentin-1PN was obtained from the transcriptome data of the frog skin transcriptome. Specific primers were designed based on sequences in the 5’- and 3’-untranslated regions (UTR) and used for PCR amplification of the complete coding region using a mixed tissue cDNA s’ obtained with other known sequences was assessed via BLAST searches (http://blast.ncbi.nlm.nih.gov/Blast.cgi). Molecular masses and theoretical isoelectric points were determined using ProtParam (https://web.expasy.org/protparam/). The presence of a signal peptide was assessed using SignalP-5.0 (https://services.healthtech.dtu.dk/services/SignalP-5.0/). Multiple sequence alignments were performed using ClustalW (http://clustalw.ddbj.nig.ac.jp/). Three-dimensional (3D) models were generated with Swiss-Model (https://swissmodel.expasy.org/). Phylogenetic and molecular evolutionary analyses were performed using MEGA X.

### Sample collection and RT-qPCR

All frogs were anaesthetised with 0.1 g/L tricaine methanesulphonate (MS-222) prior to dissection. Tissues, including the liver, heart, muscle, skin, spleen, kidney, lung, and gut, were obtained from four healthy dark-spotted frogs to analyse the expression levels of esculentin-1PN in these specific tissues.

Total RNA was extracted from the tissues using Beyozol (Beyotime, Shanghai, China). Subsequently, the PrimeScript RT reagent Kit (TaKaRa, Dalian, China) was used to synthesise first-strand cDNA. Gene-specific primers were designed based on esculentin-1PN and PnGAPDH sequences (Table 2). A qPCR assay was performed using the CFX96 real-time PCR detection system (Bio-Rad, Hercules, USA) with TB Green Premix Ex Taq (TaKaRa). The reaction mixture was incubated at 95°C for 5 min, followed by 40 amplification cycles of 30 s at 95°C, 30 s at 60°C, and 30 s at 72°C. The threshold cycle (Ct) for esculentin-1PN was normalised to PnGAPDH using the 2^−ΔΔCt^ method [47].

**Table 2:** Oligonucleotide primers sequences

Gene	Primers	Sequence (5’-3’)	Accession number
Esculentin-1PN	esculentin-1-t( +)	TGATGAAGAAGAGGGAAGCGA	OR238914
	esculentin-1-t(-)	CAACCGGCAATGTCTATCCC	
PnGAPDH	GAPDH-t( +)	ATCCCTGCTCTGAACGGAAA	FJ617544
	GAPDH-t(-)	ATTCCCTTCAGTGGTCCCTG	
MmIL-1β	IL-1β-t( +)	AGAAGCTGTGGCAGCTA	NM_008361
	IL-1β-t(-)	TGAGGTGCTGATGTACCA	
MmTNF-α	TNF-α-t( +)	GAACTGGCAGAAGAGGCACT	NM_013693
	TNF-α-t(-)	GGTCTGGGCCATAGAACTGA	
MmIL10	IL10-t( +)	GGTTGCCAAGCCTTATCGGA	NM_010548
	IL10-t(-)	ACCTGCTCCACTGCCTTGCT	
MmTGF-β	TGF-β-t( +)	TCGCTTTGTACAACAGCACC	NM_011577
	TGF-β-t(-)	ACTGCTTCCCGAATGTCTGA	
Mm18S rRNA	18S rRNA-t( +)	TTTGTTGGTTTTCGGAACTGA	NR_003278
	18S rRNA-t(-)	CGTTTATGGTCGGAACTACGA	

### Antibacterial assay

The esculentin-1PN mature peptide (GIFSKLAGKKIKNLLISGLKNVGKEVGMDVVRTGIDIAGCKIKGEC), comprising one disulfide bond, was chemically synthesised with a purity > 90% using SynPeptide and an actual molecular weight of 4.80 kDa. The mature peptide was used in subsequent experiments. The antibacterial activity of esculentin-1PN was evaluated against a variety of bacteria, including *Vibrio anguillarum*, *E. coli*, *Vibrio alginolyticus*, *Staphylococcus saprophyticus*, *Vibrio harveyi*, *Pseudomonas aeruginosa*, *Citrobacter freundii*, *Shigella flexneri*, *Aeromonas hydrophila*, *Proteus mirabilis*, *Staphylococcus warneri*, and *Listeria monocytogenes*, using a modified two-fold microdilution method, as previously described [48]. The bacterial sedimentation absorbance at 600 nm was used to evaluate the percentage inhibition of bacterial growth. The antibacterial activity was determined by the MIC, which is described here as the lowest concentration of peptide causing an OD_600_ less than 50% of the growth control (MIC_50_) [49]. The MBC was determined at the end of the experiment by taking 20 µl of the sample from the well with no visible growth, followed by serial dilution and plating.

### Lactate dehydrogenase (LDH) release assay

To assess the impact of esculentin-1PN on the integrity of bacterial cell membranes, we used an LDH Release Assay Kit (Beyotime) per the manufacturer’s instructions. *E. coli* cells, at a concentration of 1 × 10^9^ CFUs, were exposed to esculentin-1PN at concentrations of 25, 50, and 100 μg/mL and subsequently incubated at a temperature of 37 °C for a duration of 2 h. Following centrifugation, the resulting supernatant was transferred to a 96-well plate. Each well received 60 μL of the LDH detection working solution and was then incubated at 25 °C for a period of 30 min. The absorbance was subsequently measured at a wavelength of 490 nm.

### DNA degradation assay

The hydrolysis of *E. coli* (K12) genomic DNA (gDNA) by esculentin-1PN was assessed using established protocols [39]. First, gDNA was isolated and quantified from *E. coli.* The gDNA was then exposed to different esculentin-1PN concentrations (25, 50, and 100 μg/mL) and incubated for 30 min at 25 °C. Analyses were performed using a 1.0% agarose gel. gDNA was stained with 4S Green Plus Nucleic Acid Stain (Sangon, Shanghai, China), according to the instructions of the product.

### Chemotaxis assay

Per previously established protocols, a chemotaxis assay was conducted in a 24-well transwell chamber (Corning, NY, USA) [50]. Briefly, esculentin-1PN was diluted in DMEM to concentrations of 0.1, 1.0, and 10.0 μg/mL. Subsequently, 600 μl of each diluted peptide was added to the transwell chamber, which was covered with a nitrocellulose filter membrane. Next, RAW264.7 cells, a mouse leukemic monocyte/macrophage cell line, were introduced into the upper chamber and incubated at 37 °C for 4 h. The migrated cells that reached the lower chamber were stained and quantified under a magnification of 40 × 10.

### Respiratory burst assay

In RAW264.7 cells, the respiratory burst was determined by quantifying intracellular O^2−^ levels using nitro blue tetrazolium (NBT) reduction tests [51]. Before experimentation, RAW264.7 cells were subjected to pretreatment with esculentin-1PN (0.1, 1.0, or 10.0 μg/mL) for a duration of 12 h. Subsequently, the cells were washed with PBS and exposed to 0.1 μg/mL PMA. NBT was introduced to each plate and incubated at a temperature of 24 °C for a period of 1 h. Methanol was added to stop the reaction, and the cells were then thoroughly washed and dried. A wavelength of 620 nm was used to measure the optical density of the dissolved formazan.

### Inflammatory cytokine gene expression analysis in RAW264.7 cells

RAW264.7 cells were subjected to treatment with esculentin-1PN (0.1, 1.0, or 10.0 μg/mL) for a duration of 12 h. The cells were then collected, and their total RNA was extracted. The extracted RNA was then subjected to qPCR analysis following the methodology outlined in Sect. 2.3. Expression levels of IL-1β, TNF-α, TGF-β, and IL-10 were normalised to those of 18S rRNA.

### Statistical analysis

The data are presented as mean ± SEM, except for the antibacterial assay. Statistical analysis was performed using SPSS version 13.0 (SPSS Inc., Chicago, USA) with a one-way ANOVA. Statistical significance was set at a *P* value < 0.05.

### Supplementary Information

**Supplementary Material 1.**

## Data Availability

The esculentin-1PN cDNA sequence was submitted to GenBank under accession number OR238914. The datasets used and/or analysed during the current study are available from the corresponding author on reasonable request.

## Abbreviations

AMP: Antimicrobial peptide
*p*I: Isoelectric point
MIC: Minimal inhibitory concentration
MBC: Minimum bactericidal concentration
ROS: Reactive oxygen species
RNS: Reactive nitrogen species
UTR: Untranslated regions
MS-222: Tricaine methanesulphonate
Ct: Threshold cycle
LDH: Lactate dehydrogenase
gDNA: Genomic DNA
NBT: Nitro blue tetrazolium
