# Supplementary material for: An esculentin-1 homolog from a dark-spotted frog (Pelophylax nigromaculatus) possesses antibacterial and immunoregulatory properties
Source: BMC Vet Res. 2024 Apr 27;20:164. doi: 10.1186/s12917-024-04013-y (PMC11055230; doi:10.1186/s12917-024-04013-y)
Supplement: Supplementary file 1 — Supplementary Material 1. [file 12917_2024_4013_MOESM1_ESM.docx]

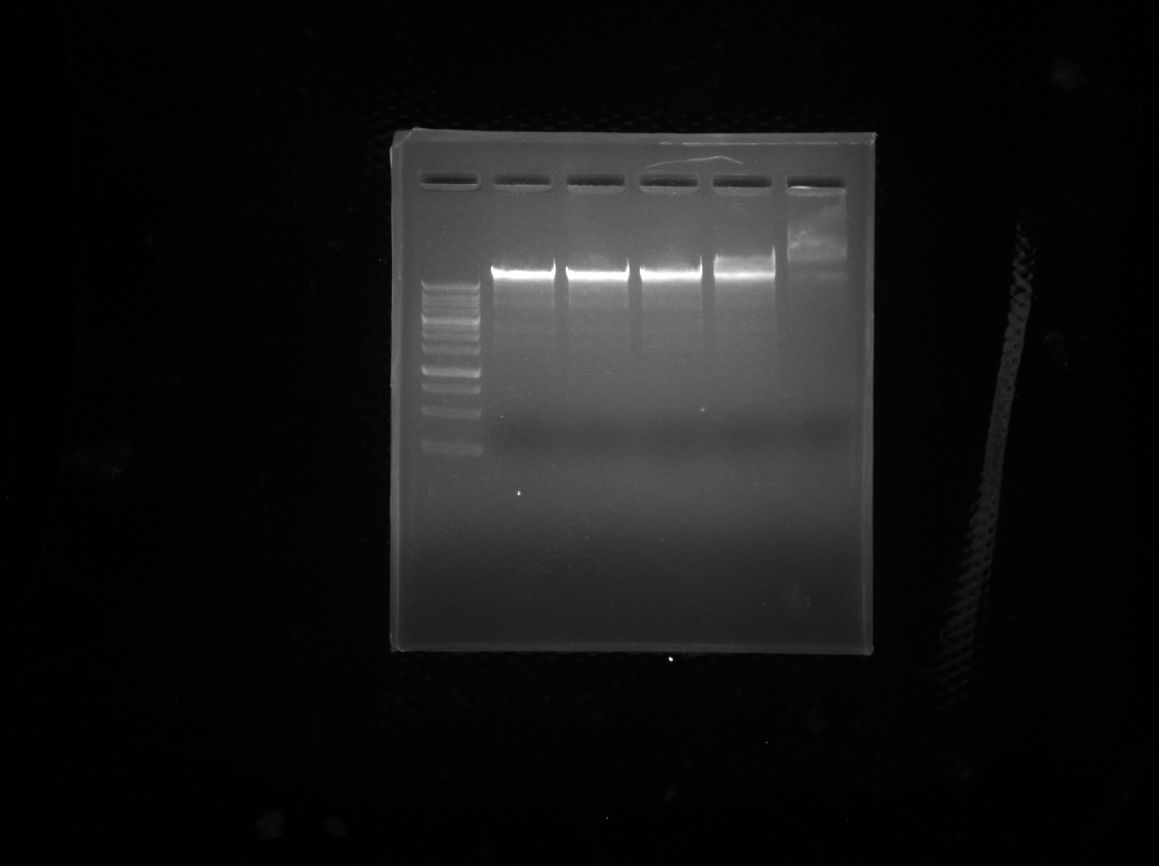


**Fig. 5.** (B) The hydrolytic activity of esculentin-1PN on the bacterial genomic DNA was assessed through electrophoresis. BSA was employed as a negative control.
